# On the Dependence of γ′ Precipitate Size in a Nickel-Based Superalloy on the Cooling Rate from Super-Solvus Temperature Heat Treatment

**DOI:** 10.3390/ma11091528

**Published:** 2018-08-24

**Authors:** Chrysanthi Papadaki, Wei Li, Alexander M. Korsunsky

**Affiliations:** 1 Multi-Beam Laboratory for Engineering Microscopy (MBLEM), Department of Engineering Science, University of Oxford, Parks Road, Oxford OX1 3PJ, UK; chrysanthi.papadaki@trinity.ox.ac.uk; 2 Rolls-Royce plc, P.O. Box 31, Derby DE24 8BJ, UK; Wei.Li@Rolls-Royce.com

**Keywords:** γ′ precipitate size, nickel superalloy, precipitation, phase transformation, kinetics, size distribution, cooling rate

## Abstract

The ability to predict the sizes of secondary and tertiary γ′ precipitate is of particular importance for the development and use of polycrystalline nickel-based superalloys in demanding applications, since the size of the precipitate exerts a strong effect on the mechanical properties. Many studies have been devoted to the development and application of sophisticated numerical models that incorporate the influence of chemical composition, concentration gradients, and interfacial properties on precipitate size and morphology. In the present study, we choose a different approach, concentrating on identifying a correlation between the mean secondary and tertiary γ′ size and the cooling rate from solution treatment temperature. The data are collected using the precipitate size distribution analysis from high-resolution scanning electron microscopy. This correlation is expressed in the form of a power law, established using experimental measurement data and rationalized using a re-derivation of McLean’s theory for precipitate growth, based on well-established thermodynamic principles. Specifically, McLean’s model is recast to consider the effect of cooling rate. The derived model captures the correlation correctly despite its simplicity, and is able to predict the mean secondary and tertiary γ′ precipitate size in a nickel superalloy, without complex modeling.

## 1. Introduction

The quest for new and improved formulations of metallic alloys resistant to mechanical loading at high temperatures has been ongoing since the middle of the 20th century, when it became apparent that these materials are crucial for power generation in the air, on land, and at sea. Nickel-based superalloys occupy a particular place in this family of materials due to their widespread use and versatility. Despite the obvious success of the existing alloy compositions, further searches for improved thermal and mechanical capabilities continue to be driven by the need to reduce emissions, while simultaneously improving performance, and hence the efficiency of aeroengines. In this context, new alloy formulations are being produced that have the potential to replace the existing alloys that reduce the weight of the components and assemblies, increase the operating temperature, or both.

The exceptionally high temperature capability of nickel-based superalloys has led to their widespread usage in structural applications in extreme environments. Their performance can largely be attributed to their microstructure, and particularly to the precipitation of the gamma prime phase (Ni_3_Al), denoted as γ′. A typical polycrystalline nickel-based superalloy consists of an A1 γ matrix with a dispersion of γ′ precipitates that possess an ordered L1_2_ structure, which may occur in three distinct distributions: primary, secondary, and tertiary. The size, morphology, and distribution of each population are determined by the applied heat treatment [1]. Most commonly, powder-processed gas turbine disc alloys are subjected to a two-stage heat treatment after thermo-mechanical processing: sub-solvus or super-solvus solution heat treatment, followed by ageing heat treatment. During super-solvus solution heat treatment, the primary γ′ formed during consolidation by hot isostatic pressing is dissolved; whereas in the case of sub-solvus solution heat treatment, it is pinned at grain boundaries, restricting grain growth. Upon cooling from the solution heat treatment, secondary and tertiary γ′ distributions nucleate, grow, and further coarsen during the subsequent ageing heat treatment [1,2].

Since the cooling rate from super-solvus solution heat treatment controls the kinetics of the secondary and tertiary γ′ precipitation, it determines to a large extent the morphology, size, and distribution of the precipitate. Primarily, the size of the precipitated γ′ is largely controlled by the cooling rate of the super-solvus temperature, as shown by previous experimental studies [3,4,5,6,7], with the average γ′ particle size decreasing with increasing cooling rates. As in many precipitation-hardened systems, the mechanical properties of nickel-based superalloys are strongly influenced by the precipitate size distribution (PSD), as it exerts control over dislocation motion. The effect of the size of the secondary and tertiary γ′ precipitates on the mechanical properties has been extensively studied [1,2,7,8,9,10]. Jackson et al. [2] observed a significant increase in hardness with decreasing average precipitate size generated by faster cooling rates, and used experiments and precipitate hardening models to rationalise this observation. They showed the existence of an optimal size of γ′ precipitates that corresponds to the transition between cutting by weakly and strongly coupled dislocations, which led to the optimum hardness and tensile strength. Additionally, for creep, the rate-controlling mechanism and its associated deformation mechanism are strongly dependent on precipitate size [8]. When the precipitate size is below a threshold value, creep deformation is dominated by γ′ cutting by matrix dislocations, as observed after microstructural examination of crept specimens. Above this critical size, Orowan bowing becomes the rate-controlling deformation mechanism [8]. Therefore, it can be concluded that creep resistance is improved significantly by a fine distribution of tertiary γ′ precipitates [9]. However, dwell crack growth resistance was shown to be enhanced by coarser γ′ precipitates, with the dependence being even stronger for tertiary γ′ [10]. Hence, the optimum combination of desired mechanical properties for specific applications can be achieved by the close control of the size of γ′ precipitates, which is dependent on the cooling rate from solution heat treatment.

By controlling the cooling rate from solution heat treatment temperature, the γ′ precipitate size can be tailored to obtain the optimum properties. To achieve this, it is of crucial importance to quantify the dependence of the average particle size on the cooling rate. This allows developing heat treatment schedules to obtain the desired precipitate size for optimum properties. In this paper, a power-law dependence of the secondary and tertiary γ′ precipitate size on the cooling rate is first observed experimentally, and then justified on the basis of thermodynamics and diffusion kinetics analysis. McLean’s model for precipitate growth [11] is recast to consider the effect of cooling rate, instead of isothermal conditions, and a simple correlation between the size of γ′ precipitates and the cooling rate is derived.

The high-temperature polycrystalline Ni-based alloy, referred to as Alloy 11 below, belongs to a family of new alloys that has been introduced recently [12]. The present study was motivated by the need to quantify the relationship between microstructure statistics (precipitate grain size) and the cooling rate from heat treatment for Alloy 11. In the process of quantifying and analysing the observed correlation, it became clear that the derived results have very broad (indeed, generic) applicability; namely, that they are relevant to all nickel-based superalloy systems, and probably also to many other precipitation-hardened alloys—a vast family of high performance engineering materials. In view of this discovery, the authors here concentrate the presentation and discussion on these matters of broad relevance, as they are likely to benefit the greatest number of researchers and practitioners in the field.

## 2. Materials and Methods

The material investigated in this study was obtained from a pancake forging of Alloy 11 (Table 1). A detailed description of the new family of alloys can be found in European Patent Specification EP2894234B1 [12]. The pancake forging was produced from Alloy 11 (with the composition provided in Table 1 below) by isothermally forging a powder isostatic pressed cylindrical compact. Miniature bar specimens with the nominal size of 40 × 2 × 1 mm^3^, extracted from the forging by electo-discharge machining, were solution treated for 1 h at 1180 °C, above the γ′ solvus of this alloy (≈1155 °C), and cooled at various rates ranging from 0.7 to 4 °C/s. Solution heat treatments with controlled cooling rates were carried out using an Electro-Thermo-Mechanical Testing (ETMT) system (Instron, High Wycombe, UK), in an argon atmosphere to minimize oxidation.

In the ETMT system, sample heating was provided by passing current directly through it, and the temperature was monitored with a thermocouple spot-welded to the center of the specimen, which was held in water cooled grips. A non-uniform temperature distribution developed along the test piece: heating current led to an approximately parabolic temperature distribution. While cooling, ETMT current was set to a value that maintains the temperature close to the required value, regulated by a proportional-integral-derivative controller (PID) software module. The middle gauge section of each specimen was used for microstructural analysis, where the temperature was monitored by the thermocouple. The solution heat treatment was followed by ageing heat treatments, for 2 h at 850 °C and then for 4 h at 800 °C.

**Figure 1:**
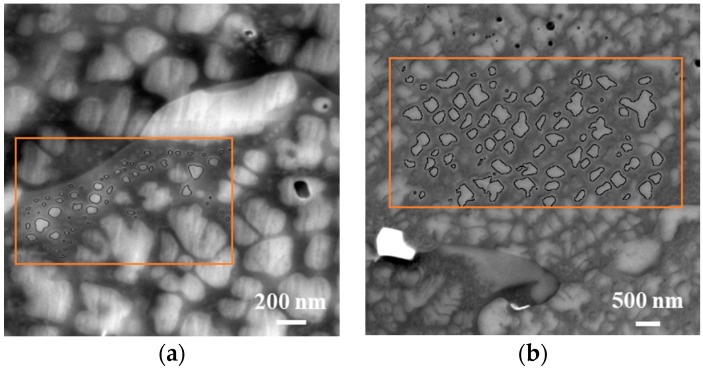
Examples of backscattered scanning electron microscopy (SEM) images used for precipitate size analysis: (**a**) for tertiary gamma prime, the magnification was ≈×170,000 and (**b**) for secondary gamma prime, the magnification used was ≈×21,000.

The samples were prepared using standard metallographic mechanical grinding and polishing techniques. A LYRA3 Focused Ion Beam-Scanning Electron Microscope (FIB-SEM) system (Tescan, Brno, Czech Republic), operating at an accelerating voltage of 10 kV, was employed to carry out microstructural examination. A back-scatter electron detector (BSE) was used to obtain good contrast between the precipitates and the matrix, allowing different populations to be distinguished. Subsequent image analysis was carried out on the SEM micrographs using ImageJ software (version 1.50e) to quantify the size of the secondary and tertiary γ′ precipitates. An appropriate number of randomly selected images with magnifications ranging between ×20,000 and ×290,000 were analysed, covering a total area of 100 µm^2^ to ensure that the γ′ size-frequency histograms generated were representative. An example is illustrated in Figure 1. To achieve statistical reliability, the diameters of a minimum of 1500 precipitates were measured for each cooling rate. The minimum dimension detected was better than 5 nm.

## 3. Results

**Figure 2:**
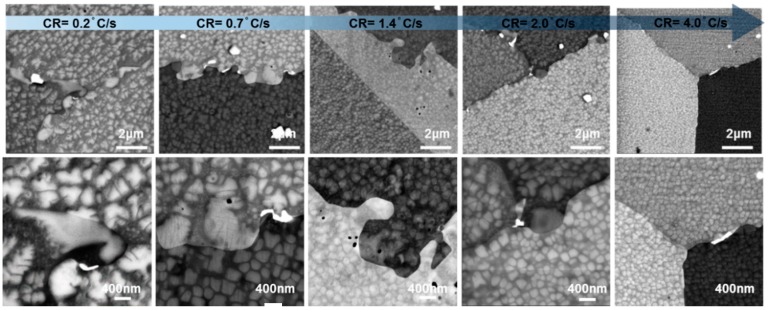
Backscattered SEM images of the microstructures developed in the samples after cooling from solution temperature at each cooling rate given. The magnification used was ≈×20,000 for the first row and ≈×50,000 for the second.

**Figure 3:**
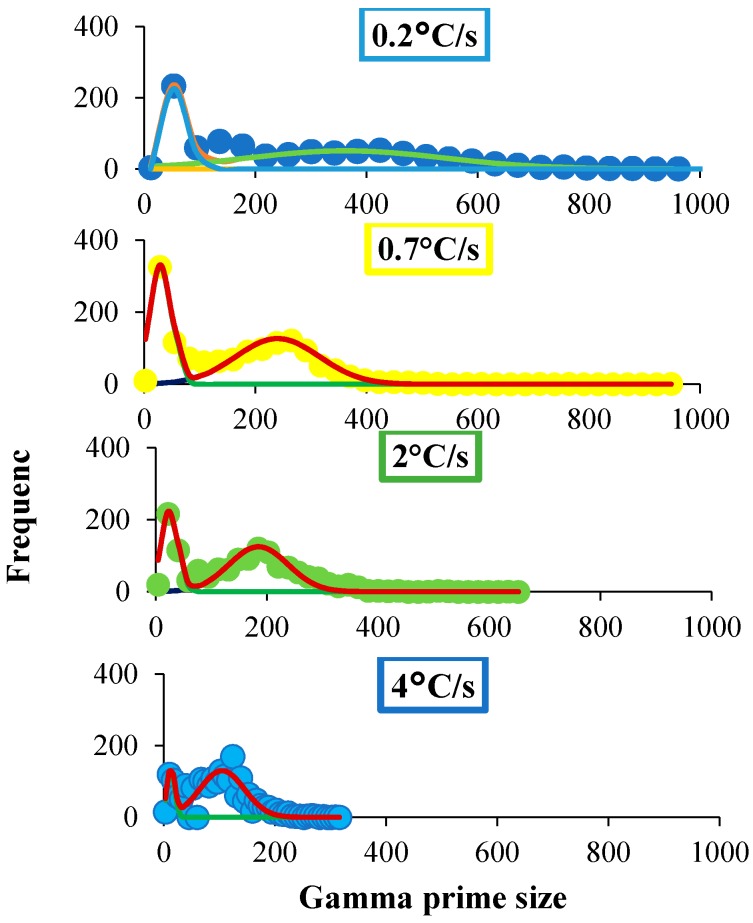
Histograms of γ′ size for each cooling rate under consideration. The continuous curves represent fits to the experimental data using Equation (1).

Figure 2 illustrates the changes that occurred in the microstructure as a function of cooling rate. The size and distribution of precipitates, as well as the morphology of grain boundaries, were observed to be strongly dependent on the cooling rate from the super-solvus solution heat treatment. For slower cooling rates, grain boundaries tended to be serrated, bowing around secondary gamma prime particles precipitated at the grain boundaries. In the grain boundary region, carbides were also present (bright phases). With faster cooling rates, there was a strong refinement of secondary and tertiary gamma prime precipitates, as expected. The results of quantitative image analysis are shown in Figure 3, which presents histograms of the probability distribution of the size of the γ′ precipitates for each cooling rate assessed. The precipitate populations appeared to conform to a bimodal size distribution, indicating the presence of secondary and tertiary γ′ particles. The continuous line denotes a fit to the data, where the left peak corresponds to the size distribution of tertiary γ′, and the right peak to that of secondary γ′ precipitates. The size distribution was fitted with a double-Gaussian function:

(1) $$y=a_{1}\;e^{-\frac{1}{2}{\left(\frac{d-{\bar{d}}_{1}}{\beta_{1}}\right)}^{2}}+a_{2}\;e^{-\frac{1}{2}{\left(\frac{d-{\bar{d}}_{2}}{\beta_{2}}\right)}^{2}}$$

where ${\bar{d}}_{1}$ and ${\bar{d}}_{2}$ are the mean diameters for tertiary and secondary γ′ precipitates in nm, respectively; $\beta_{1}$ and $\beta_{2}$ are the standard deviations; and $\alpha_{1}$ and $\alpha_{2}$ are the area ratios of the precipitates observed (Table 2).

**Figure 4:**
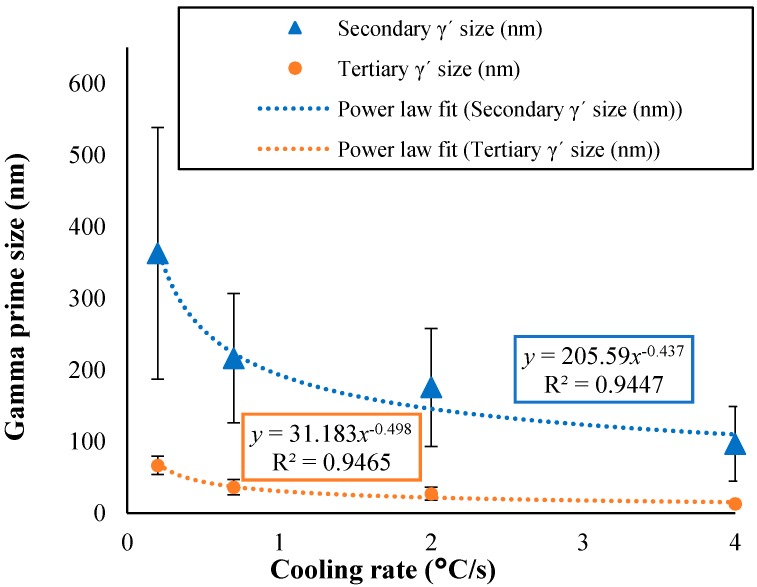
The variation in secondary and tertiary γ′ mean precipitate size with the cooling rate.

The mean precipitate size is clearly highly sensitive to the cooling rate applied. Increasing the cooling rate from the super-solvus solution heat treatment temperature resulted in significant refinement of both the secondary and tertiary γ′ particles. The variation in the average size of the γ′ particles with the cooling rate is illustrated in Figure 4. The curves shown represent a power law description of the relationship between the mean precipitate size and the cooling rate, given by:

(2) $$d=A\;c^{-n}$$

where *d* is the average precipitate diameter (nm), *c* is the cooling rate from super-solvus solution temperature (°C/s), *A* is a constant that is different between the secondary (*A* = 205.58) and tertiary (*A* = 31.183) γ′ populations, and exponent *n* also differs between the secondary (*n* = 0.437) and the tertiary (*n* = 0.498) γ′ precipitates. The measured mean precipitate size data fitted very well with the power law curve with a high correlation coefficient (R^2^) value of ~0.95 over the cooling rate range.

## 4. Derivation of the Correlation

The precipitation of the γ′ phase during cooling is driven by the local concentration gradients and involves redistribution of elements at the γ/γ′ interface. The partitioning of elements between γ and γ′ precipitates is strongly affected by the cooling rates following solution heat treatment [13], as well as the subsequent ageing thermal treatment [14]. The composition of γ and γ′ phases determines their lattice parameters and the lattice misfit between the two faces, which in turn affects the thermodynamic driving forces and kinetics of the transformation, controls the morphology and size of precipitates, and thus the mechanical properties [1,2,8,9,10]. Previous studies analytically and experimentally investigated the complex interactions between chemistry, process history, and γ′ morphology [7,13,14], and incorporated these factors into complex modelling [15]. In this approach, we deliberately did not focus our attention on the fine details of the chemical and mechanical processes that accompany the precipitate formation and population evolution to eliminate the need to calibrate for a large number of temperature-dependent material properties. The model derived provides a useful expression that captures the correlation correctly despite its simplicity. By recasting McLean’s theory to consider the effect of cooling rate rather than isothermal conditions, the simple correlation derived here is able to predict the power law dependence of secondary and tertiary γ′ particle size on the cooling rate, based on well-established thermodynamics and kinetics principles.

The power law dependence of the size of γ′ particles on the cooling rate from super-solvus solution temperature has been observed and reported previously. Furrer et al. [4] discovered a trend in the dependence of γ′ particle size on the cooling rate, and proposed a formula $d=442.5c^{-0.4605}$ to fit the experimental results. Vaunois et al. [5] used a power law relation to describe the evolution of size with the cooling rate for both sub- and super-solvus solution treatment for cast and wrought Udimet 720Li alloy. The authors identified the power law parameters *A*, *n* as 326.41, 0.2542 and 840.88, 0.3113 for the sub-solvus and super-solvus solution treatments, respectively. Later studies by Laurence et al. [6] also expressed the γ′ precipitate size evolution as a function of cooling rate from sub-solvus solution treatment for R65 alloy using a power law relationship. The parameters determined here were *A* = 364.74 and *n* = 0.3977. Moreover, Masoumi et al. [7] used a power law expression to correlate the average diameter of γ′ precipitates in AD730TM nickel-based superalloy with the cooling rate from super-solvus solution heat treatment. In this case, the power law parameters were *A* = 521 and *n* = 0.44.

In order to obtain predictions that can be compared to observations and justify the power law dependence of the precipitate size on the cooling rate, a re-derivation of McLean’s theory for mean γ′ size [11] as a function of cooling rate was used. The model developed by McLean can be used to predict the γ′ particle coarsening for nickel-based superalloys over service lifetimes using the following laws:

(3) $$r={\left[k\left(t-t_{0}\right)\right]}^{1/3}$$

(4) $$r={\left[k^{\prime }\left(t^{\prime }-t_{0}{}^{\prime }\right)\right]}^{1/2}$$

where *r* is half the mean particle size, *t* is time, and *k* is the growth rate, which conforms to the following type of temperature behavior:

(5) $$k=\frac{2.3\times 10^{15}}{T}c\;\exp\left(-\frac{32520}{T}\right)$$

where *T* is the temperature and c is the solute concentration in the matrix in wt %, i.e., the solid solubility of the diffusing element in equilibrium with a γ′ particle of infinite radius. The constant terms *t*_0_ and *t*_0_′ in the above equations are used for time-zero adjustment. For long-term exposure, the term *t*_0_ becomes insignificant and may be reasonably omitted.

McLean’s approach considers two possible mechanisms: diffusion-controlled growth and interface-controlled reaction. In his study, he showed that long-term coarsening behavior in several binary and ternary nickel-based superalloys largely follows diffusion-controlled growth behavior. However, for the initial stages of growth, scatter in experimental data generally makes it impossible to distinguish diffusion-controlled from interface-controlled growth, since data often fit *t*^1/3^ and *t*^1/2^ approximately equally well. When experimental data fail to follow one of the scaling laws of Herring [16], it is ascribed to more than one of the above mechanisms being operative over the range in sizes covered by the experiment, so that, for example, during the initial stages of growth, both the diffusion-controlled and the interface-controlled growth mechanisms are active.

The peak temperature *T*_1_ for precipitation corresponds to the maximum overall transformation rate K resulting from a competition of two opposing factors: (1) the thermodynamic driving force for nucleation, which is determined by the degree of undercooling: $\Delta T=T_{c}-T$, where *T_c_* is the equilibrium transformation temperature, so it decreases for higher temperatures; and (2) the diffusivity of the precipitating elements for the growth of the nucleus formed, which follows an Arrhenius-type temperature dependence, so it increases at higher temperatures. Therefore, K can be expressed in the following form:

(6) $$K\propto \left(T_{c}-T\right)\exp\left(-\frac{Q}{RT}\right)$$

**Figure 5:**
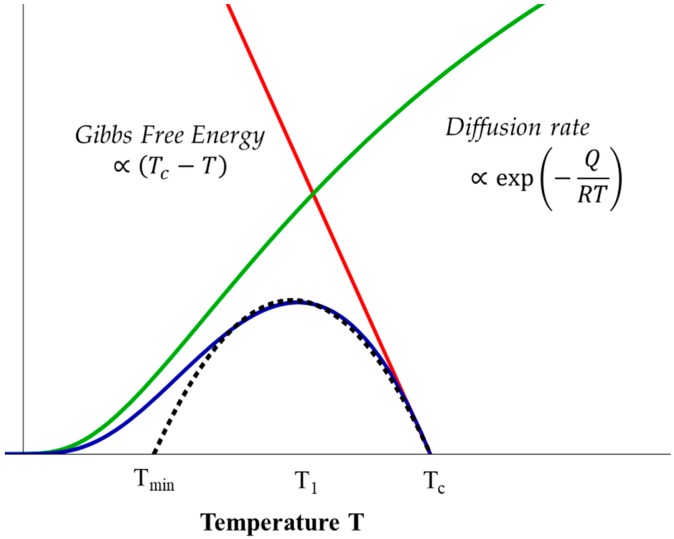
Illustration of the overall transformation rate as a function of temperature. The dashed line illustrates the parabolic approximation for the temperature dependence of the rate of the overall transformation. *T_min_*: Assumed to be the minimum temperature below which the diffusivity is too slow for the transformation. *T_1_*: Critical temperature for maximum overall transformation rate. *T_c_*: Equilibrium transformation temperature for nucleation.

Thus, the maximum of the overall transformation rate occurs at an intermediate critical temperature *T*_1_. For the purposes of simplified analysis, a convenient approximation of the temperature dependence of the overall transformation rate could be used in the form of a parabolic function, which reaches a maximum when *T = T*_1_ (dashed line in Figure 5). The normalised temperature *θ* is introduced as:

(7) $$\theta =\frac{T_{c}-T}{\Delta Τ}$$

where $\Delta T=T_{c}-T$. The overall transformation rate can be written approximately in the form:

(8) $$K\left(\theta \right)=1-{\left(1-\theta \right)}^{2}\Rightarrow K\left(\theta \right)=\theta \left(2-\theta \right)$$

Assuming the cooling rate from the solution temperature to be constant:

(9) $$c=\frac{d\theta }{dt}\Rightarrow \theta =ct$$

Therefore, the overall transformation rate can be expressed as:

(10) K(t)=ct(2−ct)

If the time-zero adjustment term *t*_0_ is ignored, McLean’s model for growth can be re-cast in the following form:

(11) $$r={\left(k\;t\right)}^{1/3}\;\Rightarrow \;r^{3}=k\;t$$

(12) $$r={\left(k^{\prime }\;t\right)}^{1/2}\underset{\;}{\Rightarrow }r^{2}=k^{\prime }\;t$$

So, the combination of the above Equations (11) and (12) produces:

(13) $$r^{\beta }=Kt$$

(14) $$\Leftrightarrow \dot{r}=\frac{1}{\beta }Kr^{-\left(\beta -1\right)},\;\beta =2÷3$$

where K is the rate of the overall transformation, which is dependent on temperature, as explained above. Substituting K from Equation (10) into Equation (14) gives:

(15) $$\dot{r}=\frac{1}{\beta }ct\left(2-ct\right)r^{-\left(\beta -1\right)}$$

Separating variables and integrating the above equation gives:

(16) $$\int_{0}^{R}r^{\left(\beta -1\right)}dr=\int_{0}^{t_{max}}\frac{1}{\beta }ct\left(2-ct\right)dt$$

where *t_max_* is the time when precipitate growth terminates (in the simplified description adopted here), i.e., when temperature reaches *T_min_*, or *θ* = 2. This allows the maximum precipitate size *R* achieved after time *t_max_* to be expressed as:

(17) $$\frac{R^{\beta }}{\beta }=\frac{4}{3\beta c}\Rightarrow R\propto c^{-1/\beta }$$

Considering that β=2÷3, we concluded that the mean particle size exhibits a power law dependence on the cooling rate that can be expressed in the following form:

(18) $$R=A\;c^{-n}\{\begin{array}{c} n=1/2\overset{\;}{\to }\mathrm{interface}\;\mathrm{controlled}\;\mathrm{growth}\\ n=1/3\overset{\;}{\to }\mathrm{diffusion}\;\mathrm{controlled}\;\mathrm{growth} \end{array}$$

## 5. Discussion

It was apparent that the size of precipitates observed by quantitative image analysis (Figure 4) was in good agreement with the predictions of the model described above, indicating that the growth of secondary and tertiary γ′ is controlled by a combination of diffusion-controlled and the interface-controlled mechanisms. As indicated in Equation (18), the power law exponent n indicates the mechanism for growth. The value of *n* typically lies between the two bounds, whereas the proximity to one of the extreme values reflects the dominance of a particular growth mode. Parameter *A* in Equation (18) is a constant that takes different values for the secondary and tertiary gamma prime populations. Accordingly, it is likely to be different for different alloy systems. In particular, for the case of tertiary γ′ precipitates n=0.498, suggesting that the interface-controlled growth mechanism is predominant. For tertiary γ′ precipitates, the surface over volume ratio is high, promoting the growth to be interface-controlled.

### The Effect of Ageing Heat Treatment

In the present study, the samples were exposed to consistent ageing heat treatment after cooling from solution temperature at different rates. Various theories have been developed to describe the effect of ageing and long-term isothermal exposure to the precipitate distribution function. The Lifshitz-Slyozof-Wagner (LSW) theory was developed to model precipitate growth and has been applied to the diffusion-controlled Ostwald ripening of the γ′ precipitate particles in nickel-based superalloys [17]. According to the classical LSW theory, the average precipitate radius *r* is predicted to increase with time, such that *r*^3^ ∝ *t*, whereas the precipitate distribution function is declared to be time independent: the particle radius distribution was found to be self-similar under the scaling of the average particle size. Hopgood et al. [18] compared the experimentally determined values for the precipitate size and the theoretical distributions predicted by LSW theory. They found that, after short aging times, there was good connection between the shapes of the experimental distributions and those predicted.

McLean [11] examined the precipitate size distributions after isothermal exposure for binary, ternary, and commercial nickel-based superalloys reported by previous studies. The literature data were analyzed on the basis of equivalent time (*t_eq_*) calculated for a chosen equivalent temperature (*T_eq_*). By plotting the precipitate size distributions at the beginning of the process, after considerable growth and at the end of *t_eq_*, the error introduced by regarding the precipitate size distribution as steady state was minor, as the change in distribution was very sluggish compared to the growth of the average precipitate size.

From the aforementioned studies [11,17,18], it can be inferred that, after the ageing heat treatment, the size distribution of the γ′ precipitates would shift toward a larger mean size, but the overall pattern would remain unaltered. The effect of ageing heat treatment on this pattern may be minor compared to the effect of the cooling rate, and thus the observed pattern is fully attributed to the cooling rate from the super-solvus solution heat treatment.

## 6. Summary

To summarize, in this study, changes in terms of size of γ′ precipitates in a polycrystalline nickel-base superalloy, Alloy 11, were investigated after super-solvus heat treatments with various controlled cooling rates. Through quantitative image analysis, we observed that the variation in secondary and tertiary γ′ precipitate size had a power law dependence on the cooling rate from super-solvus solution temperature. This trend was interpreted by a simplified model based on the McLean theory of growth of γ′ in nickel alloys. The primary contribution of this model is the identification of a simple correlation between the γ′ precipitate size and the cooling rate, with which useful insights and predictions can be obtained, even without knowing the large number of temperature-dependent material properties required for complex modelling. During cooling form the solution temperature, the growth of secondary γ′ is controlled by a combination of diffusion-controlled and interface-controlled mechanisms, and the growth of tertiary γ′ is primarily controlled by the interface-controlled mechanism. The results obtained from this study contribute to the better understanding of the growth mechanism of γ′ precipitates and can be used to design the heat treatment applied by choosing the cooling rate after the solution heat treatment to tailor the particle precipitate size for the optimum combination of the desired properties.

## Figures and Tables

**Table 1:** Chemical composition of Alloy 11 (wt %) [12].

Ni	Co	Cr	Ta	W	Al	Ti	Mo	Nb	Fe	Mn	Si	Zr	C	Bo
bal	15.06	12.69	4.77	3.22	3.16	2.84	2.14	1.44	0.95	0.48	0.47	0.057	0.027	0.023

**Table 2:** Parameters for the double-Gaussian fit for each cooling rate.

Cooling Rate (°C/s)	${\bar{d}}_{1}$	${\bar{d}}_{2}$	$\beta_{1}$	$\beta_{2}$	$\alpha_{1}$	$\alpha_{2}$	R^2^
0.2	66.98	362.8	12.79	175.5	414.2	50.94	0.9757
0.7	36.5	216.4	10.72	90.26	381.7	119.6	0.989
2	27.34	175.8	9.224	82.34	236.7	97.95	0.9854
4	13.37	96.83	1.806	52.17	116.8	116.1	0.8986
